# Comprehensive characterisation of immunogenic cell death in melanoma revealing the association with prognosis and tumor immune microenvironment

**DOI:** 10.3389/fimmu.2022.998653

**Published:** 2022-09-23

**Authors:** Jie Ren, Jiaqi Yang, Song Na, Yiqian Wang, Linyun Zhang, Jinkui Wang, Jiwei Liu

**Affiliations:** ^1^ Department of Oncology, The First Affiliated Hospital of Dalian Medical University, Dalian, China; ^2^ Department of Orthopedics, First Affiliated Hospital of Dalian Medical University, Dalian, China; ^3^ Emergency Intensive Care Unit, Affiliated Zhongshan Hospital of Dalian University, Dalian, China; ^4^ Department of Radiotherapy, The First Affiliated Hospital of Dalian Medical University, Dalian, China; ^5^ Department of Neurosurgery, First Affiliated Hospital of Dalian Medical University, Dalian, China; ^6^ Department of Plastic Surgery, First Affiliated Hospital of Dalian Medical University, Dalian, China

**Keywords:** melanoma, immunogenic cell death, pan-cancer analysis, tumor immune microenvironment, prognosis

## Abstract

Increasing evidence has highlighted the critical functions of immunogenic cell death (ICD) within many tumors. However, the therapeutic possibilities and mechanism of utilizing ICD in melanoma are still not well investigated. Melanoma samples involved in our study were acquired from The Cancer Genome Atlas (TCGA) and the Gene Expression Omnibus (GEO) databases. First, pan-cancer analysis of ICD systematically revealed its expression characteristics, prognostic values, mutation information, methylation level, pathway regulation relationship in multiple human cancers. The non-negative matrix factorization clustering was utilized to separate the TCGA-melanoma samples into two subtypes (i.e. C1 and C2) with different prognosis and immune microenvironment based on the expression traits of ICD. Then, LASSO-Cox regression analysis was utilized to determine an ICD-dependent risk signature (ICDRS) based on the differentially expressed genes (DEGs) between the two subtypes. Principal component analysis and t-distributed stochastic neighbor embedding analysis of ICDRS showed that high- and low-risk subpopulations could be clearly distinguished. Survival analysis and ROC curves in the training, internal validation, and external validation cohorts highlighted the accurate prognosis evaluation of ICDRS. The obvious discrepancies of immune microenvironment between the different risk populations might be responsible for the different prognoses of patients with melanoma. These findings revealed the close association of ICD with prognosis and tumor immune microenvironment. More importantly, ICDRS-based immunotherapy response and targeted drug prediction might be beneficial to different risk subpopulations of patients with melanoma. The innotative ICDRS could function as a marker to determine the prognosis and tumor immune microenvironment in melanoma. This will aid in patient classification for individualized melanoma treatment.

## Background

The most severe type of skin cancer is melanoma, which is caused by a malignancy of melanocytes (1). It is late diagnosis that leads to the poor prognosis in melanoma (2). Although typical therapies such as surgical excision (3), immunotherapy (4), gene therapy (5) are wildly used for melanoma patients, the mortality of melanoma has increased steadily in the last decades, which results in public health problems (6). Hence, there is need to come up with sensitive approaches for accurate assessment of clinical outcomes of melanoma patients, facilitating the development of precision medicine.

In recent years, most immune system components have been notified to be linked to melanoma’s genesis and progression (7, 8). Currently, PD-1, PD-L1, and CTLA-4 inhibitors which are examples of immunotherapy medications are applied in melanoma (9–11). However, these treatments are only effective in a small number of patients, with a vast number of patients having a restricted or non-existent response to treatment, particularly as the melanoma progresses. As a result, in order to investigate the potential predictive usefulness of immune and immune-related indicators, extensive investigations of the relationship between immune and melanoma are required.

The Nomenclature Committee on Cell Death (NCCD) has developed recommendations for defining and interpreting cell death from morphological, biochemical, and functional viewpoints over the last decade (12). Immunogenic cell death (ICD) is a distinct sort of cell death produced by a variety of anticancer treatment modalities, such as radiotherapy and chemotherapeutic medicines. In immunocompetent hosts, it entails activating the immune system against malignancy. ICD is followed by the exposure and generation of various molecular patterns linked to damage, which offer a strong adjuvanticity to dying cancer cells by favoring antigen-presenting cell recruitment and activation (13–15). Improving the immunogenicity of tumor cells by inducing ICD is a crucial strategy for improving cancer immunotherapy (16). However, the therapeutic possibilities and mechanism of utilizing ICD in melanoma are still not well investigated.

In this research, two subtypes with different prognosis and immune environment in melanoma were identified on the basis of ICD-related genes and an ICD-dependent risk signature (ICDRS) was created with the differentially expressed genes (DEGs) between the two melanoma subtypes. Additionally, the relation between the signature, prognosis and immune were further analyzed. The findings in the study illustrated that a novel ICDRS might be utilized as a helpful marker for the prognostic prediction and immune environment assessment in melanoma.

## Materials and methods

### Data collection

The Cancer Genome Atlas (TCGA) system was established in 2006 by the National Human Genome Institute and the National Cancer Institute with the purpose to map cancer genes, understand cancer’s potential pathways, and improve the ability of preventing the advancement of cancer, making precise diagnoses, and curing cancer (https://portal.gdc.cancer.gov/). High-throughput microarray and next-generation sequencing gene function data sets are archived in the Gene Expression Omnibus (GEO), a public database that is accessible worldwide. In the current study, the TCGA database was used to gather mRNA expression, clinical features, single nucleotide variation (SNV), copy number variation (CNV) and methylation data of pan-cancer (17, 18). In addition, GEO database was also searched to acquire mRNA expression profiles and corresponding clinical characteristics of melanoma transcriptome (19–21). ICD-related genes were identified based on the literature (22). Common immune checkpoint genes (ICGs) were identified from the review (23). The ‘c2.cp.kegg.v7.4.symbols.gmt’ file received from the Molecular Signatures Database (MSigDB) was used to identify immune-related pathway genes (MSigDB) (24–26).

### Data procession

To find intersecting genes, the intersection of melanoma mRNA expression matrix from TCGA and melanoma mRNA expression matrix from GEO were taken. Data about crossing genes’ expression from the TCGA and GEO were transformed to log2(x + 1) form and batch normalized by conducting the “ComBat” function in the “sva” package in R. The transcriptome data of the intersecting genes in TCGA and GEO datasets were merged with corresponding clinical data, respectively.

### Pan-cancer analysis

In recent years, various studies have been conducted to investigate the association between ICD-related genes and malignancies. Nonetheless, the prognostic effect, expression level, CNV, SNV and methylation of ICD-related genes in different types of cancers are poorly summarized. Thus, a pan-cancer assessment about these factors of ICD-related genes was carried out using the similar methods as the previous studies (27–32). Fold change of expression of ICD-related genes in pan-cancer was assessed. Univariate Cox regression analysis was performed to determine the ICD-related genes’ prognostic importance in distinct malignancies. CNV amplification and CNV deletion were evaluated. SNV of each gene was accumulated and the mutation frequency were calculated as follows: samples with SNV/all samples. The SNV type was also summed up. The pan-cancer methylation variation compared with normal tissue was analyzed. Additionally, for unveiling immune-related pathways affected by ICD-related genes, ICD scores in each sample of each cancer were computed through single sample gene set enrichment analysis (ssGSEA). Samples with the bottom and top 30% of ICD scores were selected into two groups respectively. Then, gene set enrichment analysis (GSEA) and the transcriptome were utilized to explore the difference of immune-related pathway activities caused by ICD-related difference between high-ICD and low-ICD groups. R and TBTools were used to conduct all of these analyses (33).

### NMF clustering identification of two subtypes of melanoma

The mRNA expression matrix of ICD-related genes in TCGA dataset was collected to perform non-negative matrix factorization (NMF) clustering with the adjusted number of clusters as 2-10 by utilizing the “NMF” package in R (34). The standard “brunet” option was selected, and 100 iterations were performed. The most appropriate clustering number was determined based on the NMF rank surveys and discrimination between different cluster subtypes (35).

### Comparison of the clinical traits, survival status, tumor immune microenvironment, and gene expression levels between different cluster subtypes

The fisher test was employed to the compositional discrepancies of clinical traits in different subtypes. Kaplan–Meier analyses were conducted to investigate the differences of disease specific survival (DSS), overall survival (OS), and progression free interval (PFI) in different subtypes. The wilcox test was employed to investigate the discrepancies of tumor immune microenvironment in different cluster subtypes after computing the ImmuneScore and TumorPurity of each melanoma sample by utilizing the “estimate” package in R (36). The CIBERSORT approach was employed to compare the infiltration composition of 22 immune cells in each melanoma sample (37, 38). The wilcox test was then implemented to investigate the discrepancy in the immune cell infiltration and the ICG expression levels between different subtypes (39). The ‘limma’ package in R was utilized to identify the ICD-dependent DEGs (ICD-DEGs) between the two melanoma subtypes and FDR < 0.05 and | log 2 fold-change (FC) | > 1 were used as screening criteria.

### Development and verification of a risk signature based on ICD- DEGs

Melanoma samples from the TCGA dataset with full transcriptome and survival data were randomly separated into two cohorts: train and test1. Following that, all TCGA samples were set as test2 cohort, whereas every GEO sample was included in test3 cohort.

In train cohort, univariate Cox regression analysis was carried out for distinguishing prognostic ICD-DEGs (screening criteria: p < 0.05). Secondly, to minimize over-fitting and choose relevant variables among the prognostic ICD-DEGs, least absolute shrinkage and selection operator (LASSO) regression analysis was employed. After that, multivariate Cox proportional hazards regression analysis was conducted for identifying an ICD-dependent risk signature (ICDRS) and the risk score of each sample was determined under the help of the “predict” function in R. Following the calculation of each sample’s risk score in the train cohort, samples were stratified into low- and high-risk subpopulations depending on the median value. Then, based on the median risk score of the train cohort, the melanoma patients in the three test cohorts were all categorized into high- and low-risk subpopulations. The following analyses were carried out in the train and three test cohorts for ICDRS creation, external validation, and internal validation: (1) the use of principal component analysis (PCA) and t-distributed stochastic neighbor embedding (t-SNE) to visualize sample classification; (2) the use of Kaplan-Meier analysis to investigate the discrepancies of survival status in high- and low-risk subpopulations; (3) the use of the ‘pheatmap’ R package to display the expression levels of the genes in the ICDRS; (4) the use of the wilcox test for investigating the variations of tumor immune microenvironment in low- and high-risk subpopulations after computing the ImmuneScore and TumorPurity of each melanoma sample utilizing transcriptome data the “estimate” package in R; (5) the use of Pearson correlation analysis to illustrate the correlation between ImmuneScore and TumorPurity and risk score; (6) the use of a time-dependent receiver operating characteristic (ROC) curve to verify the ICDRS diagnostic values for 0.5-year, 1-year, and 2-year survival rates utilizing the ‘survivalROC’ package in R. (7) combined application of survival analysis, time-dependent ROC curve, and C-index to highlight the accuracy of our signature comparing with another three well-established signatures [a ferroptosis-related signature recognized by Zeng et al. (40), a metabolism-related signature recognized by Deng et al. (41), and a pyroptosis-related signature recognized by Wu et al. (42)].

### The ICDRS-based immune-related discrepancies in all the four cohorts

After the investigation about the discrepancies of the tumor immune microenvironment between the low- and high-risk subpopulations, the ICDRS-depend immune-related discrepancies were studied in depth. First, the different expression analysis of ICGs and ICD-related genes between low- and high-risk subpopulations was conducted by utilizing the wilcox test. Then, the varied infiltration of immune cells between low- and high-risk subpopulations was analyzed after calculating the infiltration composition of 22 immune cells in every melanoma sample according to CIBERSORT algorithm (37, 43). In addition, the activities of immune-related pathways were compared with the help of the wilcox test after evaluating the activities of these pathways according to the transcriptome data by the single sample gene set enrichment analysis (ssGSEA) in R (18, 44, 45).

### The correlation analysis between ICDRS-based risk score and immune-related indicators in all the four cohorts

All the statistically different immune-related indicators in all cohorts concurrently were studied deeply in the following analysis. It is Pearson correlation analysis that was performed for illustrating the relationship between ICDRS-based risk score and immune-related indicators including the proportion of immune cells existing in the tumor immune environment, the expression of ICGs and ICD-related genes, and the immune-related pathway scores.

### Prediction of immunotherapy response and potential drugs for melanoma treatment based on ICDRS

Immunotherapy is wildly applied in melanoma. To distinguish patients more suitable for immune checkpoint inhibitor (ICI) treatment, the Cancer Immunome Atlas (TCIA), a database helping predict immunotherapy response, was searched to downloaded immunophenoscores (IPS) of melanoma samples in TCGA (46, 47). Subsequently, wilcox.test was utilized to compared IPS between low- and high-risk subpopulations in the three cohorts derived from TCGA. Of note, IPS is a satisfied predictor for anti-PD-1 and anti-CTLA-4 therapies. In order to investigate the potential drugs for melanoma patients, DEGs between low- and high-risk subpopulations were additionally explored utilizing the ‘limma’ in R and those DEGs which expressed highly in high-risk subpopulation were identified with the filtering parameters were FDR < 0.05 and log2 FC > 1. Then CMap database (https://clue.io/COMMAND) was applied to predict potential drugs which targeted highly expressed DEGs in all the four cohorts respectively.

## Results

### Data procession

**Figure 1:**
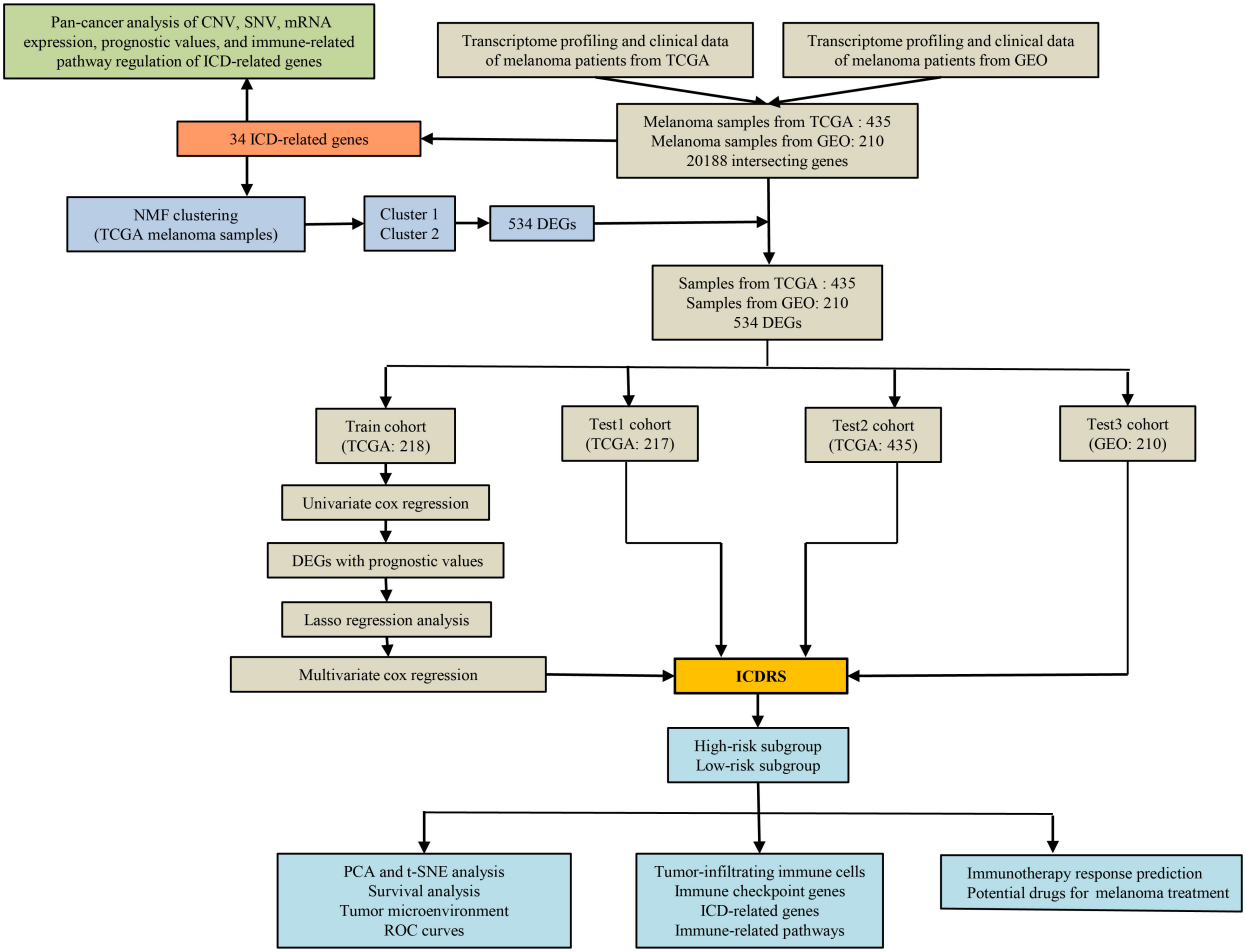
The present study’s workflow.

**Figure 1** shows a flow chart with a summary of the research process. The analysis includes 472 melanoma samples from the TCGA database and an additional 214 melanoma samples (GSE65904) from the GEO database. A total of 20,188 common genes were found after all of the genes from the TCGA and GEO datasets were intersected. Notably, 37 TCGA melanoma samples and 4 GEO melanoma samples were omitted because their survival data was incomplete. Totally, the mRNA expression data and survival data of 435 TCGA melanoma samples and 210 GEO melanoma samples were merged respectively. For the following research, 34 ICD-related genes were included.

### ICD-related genes’ mRNA expression and prognostic significance across cancer types

**Figure 2:**
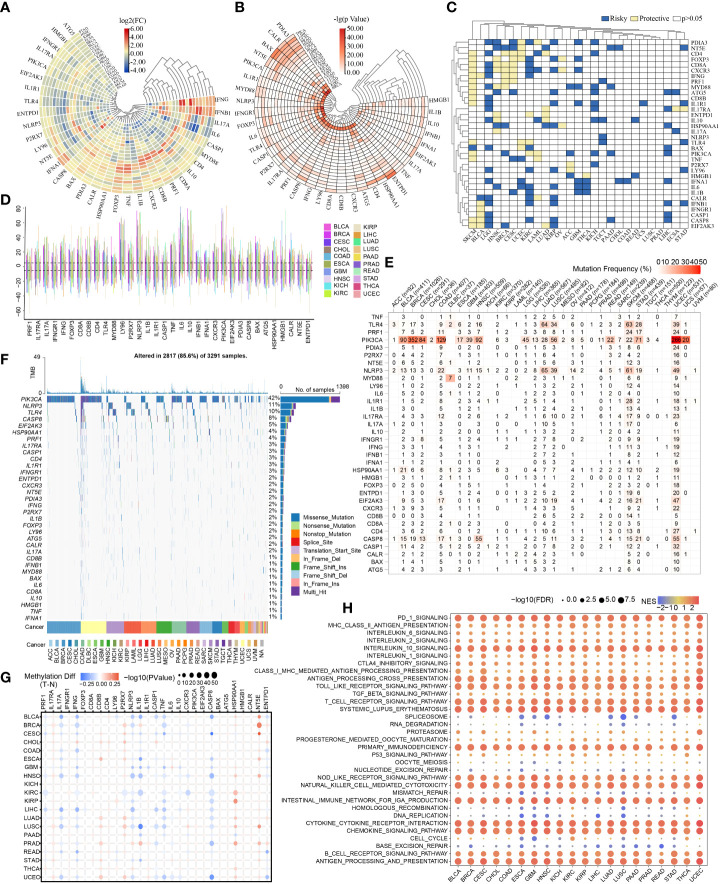
Pan-cancer overview of ICD-related genes. The discrepancies in expression levels of ICD-related genes across pan-cancer and corresponding paracancerous tissues **(A)**: log2(FC), **(B)**: -lg(pValue)). **(C)** Survival landscape of ICD-related genes across cancer types. **(D)** CNV of ICD-related genes in various cancers. **(E, F)** SNV frequencies and types in pan-cancer. **(G)** Methylation variation of ICD-related genes in pan-cancer. **(H)** Immune-related pathways affected by ICD-related genes (The redder the color, the higher the normalized enrichment score (NES); the larger the dot, the lower the corrected p-value).

First, **Figure 2A** shows the levels of mRNA expression of ICD-related genes. In the heat map, IFNG indicated a clearly elevated expression in CESC, KIRC, and GBM. IFNB1 showed a markedly elevated expression in BRCA and BLCA, while IL6 showed an obviously decreasing expression in BRCA and BLCA. To more vividly demonstrate the importance of variance in mRNA expression levels, a heat map displaying -lg (pValue) was constructed. The more intense the change of mRNA expression in corresponding tumor, the redder the color. (**Figure 2B**). The results of univariate cox regression analysis between the mRNA expression and OS distinguished risky ICD-related genes (HR>1, p<0.05) and protective ICD-related genes (HR<1, p<0.05). Of note, CD4, FOXP3, CD8A, CXCR3, IFNG, PRF1, MYD88, ATG5, CD8B, IL1R1, TLR4, PIK3CA, TNF, CASP8, and EIF2AK3 showed protective function in SKCM (**Figure 2C**).

### CNV, SNV, methylation of ICD-related genes and immune-related pathways affected by ICD-related genes in different types of cancers

The CNV, SNV and methylation existed in various cancers. The summary of pan-cancer CNV suggested CNV occurred in various cancers at high frequencies (>5%) (**Figure 2D**). The SNV states of ICD-related genes were evident and attractive in UCEC. And the PIK3CA showed higher SNV in BRCA, COAD, and UCEC. The mutation frequency of PIK3CA SNV in UCEC reached nearly 50% (**Figure 2E**). Exactly, the SNV types of PIK3CA were mainly Missense_Mutation (**Figure 2F**). Of note, the PIK3CA methylation made no sense in most cancers (**Figure 2G**). Indeed, ICD-related genes might correlate to many immune-related pathways which were shown in **Figure 2H**.

### NMF clustering identifying of two melanoma subtypes

**Figure 3:**
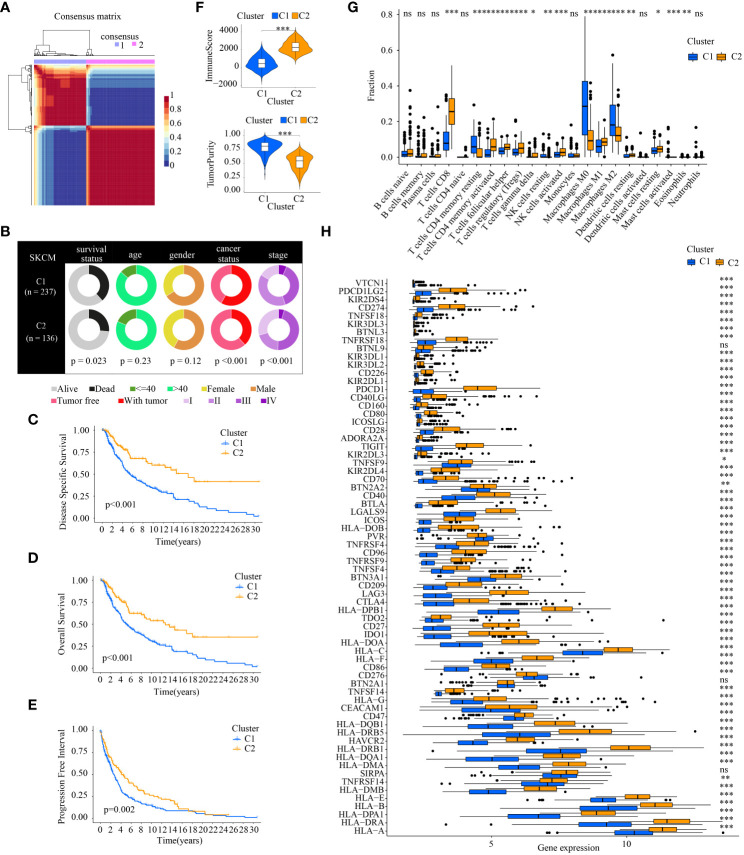
Comparison of the clinical traits, survival status, tumor immune microenvironment between different cluster subtypes obtained by NMF clustering. **(A)** The optimal clustering number of 2; **(B)** Pie charts illustrating the clinicopathologic factors in the two molecular subtypes; **(C–E)** Kaplan–Meier analyses (DSS, OS and PFI) based on the two molecular subtypes; **(F)** Comparison of TME components; **(G)** Discrepancy analysis of tumor-infiltrating immune cells in different subtypes; and **(H)** Differential expression analysis of 68 immune checkpoints genes between two molecular subtypes.(* indicates p <0.05; ** indicates p < 0.01; *** indicates p < 0.001; ns: p > 0.05).

According to the ICD-related genes’ expression matrix, NMF clustering was conducted and the optimal clustering number of 2 was selected (**Figure 3A**). The compositional differences of clinical traits between cluster1 and cluster2 suggests that the two subtypes differed statistically in many aspects such as survival status, cancer status, and tumor stage (**Figure 3B**). As for the different survival status in the two clusters, samples in cluster2 had better DSS, OS, and PFI (**Figures 3C–E**).

The tumor immune microenvironment is also statistically different. The higher ImmuneScore, which is correlated with immune components, existed in C2. The TumorPurity in C2 is worse than those in C1 (**Figure 3F**). Of note, the tumor-infiltrating immune cells in different subtypes showed different percentages. There were more anti-tumor immune cells in C2 such as CD8^+^ T cells, activated CD4^+^ T memory cells, activated NK cells, and M1 macrophages. As for cancer-promoting immune cells, M2 macrophages are downregulated in C2 (**Figure 3G**). ICGs also displayed discrepancies in the two cluster subtypes. Almost all the ICGs had a higher expression in C2. It is noteworthy that common immunotherapy targets including PD-1(PDCD1), PD-L1(CD274), and CTLA4 were highly expressed in C2 (**Figure 3H**).

### Investigation of ICD-DEGs and construction of an ICDRS

In view of the statistically different survival status and tumor immune microenvironment in the two cluster subtypes, the two clusters can be differentiated from each other. Then 534 ICD-DEGs between cluster1 and cluster2 were identified (**Supplementary Figure 1**). The findings of the univariate Cox regression analysis revealed that 237 of the 534 ICD-DEGs might be used as prognostic predictors. Subsequently, collinearity among the 237 ICD-DEGs was eliminated and over-fitting of the prognostic signature was avoided through LASSO regression analysis (**Supplementary Figures 2A, B**). 3 ICD-DEGs were selected for further multivariate Cox regression analysis (**Supplementary Figure 2C**). Finally, an ICDRS was developed using multivariate Cox proportional hazards regression analysis incorporating 3 ICD-DEGs (i.e., GBP2, THBS4, and APOBEC3G). The “predict” function in R was applied to calculate risk score of each patient with melanoma in all the four cohorts, and samples were separated into high- and low-risk subpopulations using the median risk score of 1.0342095 in train as cutoff value.

**Figure 4:**
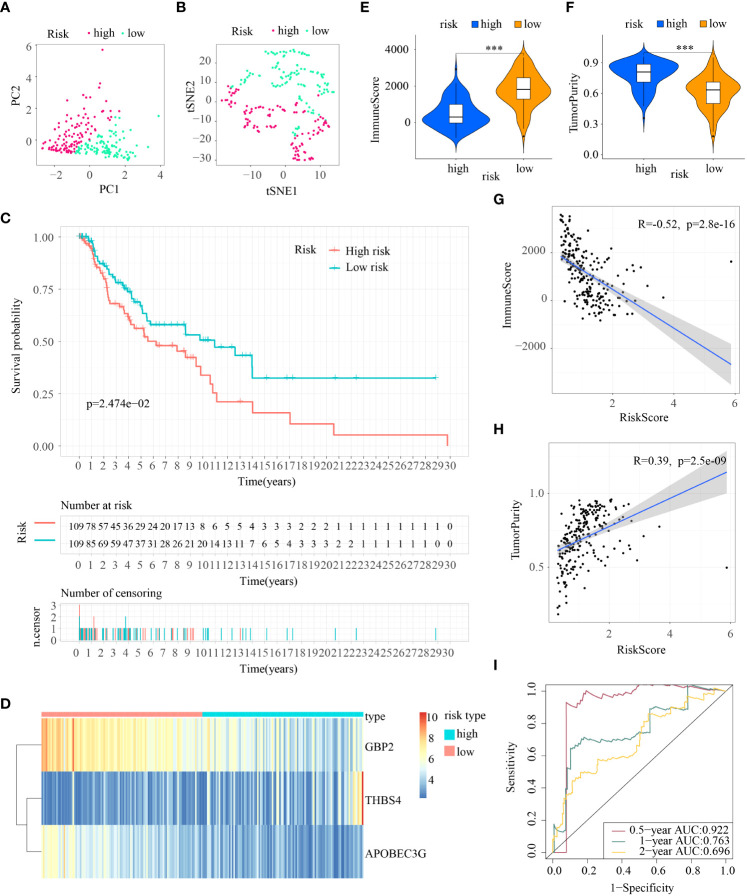
Construction of ICDRS in the train cohort. **(A, B)** PCA and t-SNE analysis illustrated an excellent clustering performance of the ICDRS-based risk score; **(C)** Kaplan–Meier survival curves for the overall survival of two risk groups in the train cohort; **(D)** Distribution pattern of the expression levels of the 3 genes in the train cohort; **(E, F)** Comparing TME components in train cohort (*** indicates p < 0.001); **(G)** The correlation between ImmuneScore and risk score; **(H)** The correlation between TumorPurity and risk score; **(I)** AUC values of ROC curves in the train cohort.

PCA and T-SNE were then conducted to determine the overall distribution of melanoma samples in low- and high-risk subpopulations. The patients within the two subpopulations can be effectively differentiated (**Figures 4A, B**). The survival analysis illustrated samples in the two subpopulations correspond to different survival status: OS rates were lower in the high-risk subpopulation (p < 0.05) (**Figure 4C**). Then levels of expression of these three ICD-DEGs in ICDRS are shown by a heatmap: GBP2 and APOBEC3G in high-risk subpopulation had lower levels of expression while THBS4 had higher expression in high-risk subpopulation compared with low-risk subpopulation (**Figure 4D**). Moreover, the tumor immune microenvironment was also statistically different in low- and high-risk subpopulations: patients with high-risk exhibited decreased levels of ImmuneScore but showed increased levels of TumorPurity compared with low-risk patients (**Figures 4E, F**); in addition, the ImmuneScore indicated a highly adverse correlation with risk score (R=-0.52, P=2.8e-16) while the TumorPurity indicated a moderately positive correlation with risk score (R=0.39, P=2.5e-09) (**Figures 4G, H**). Moreover, the ROC curves’ area under the curve (AUC) values are 0.922, 0.763, and 0.696 for 0.5-, 1-, and 2-year survival (**Figure 4I**).

### Internal and external validation of the ICDRS in melanoma

**Figure 5:**
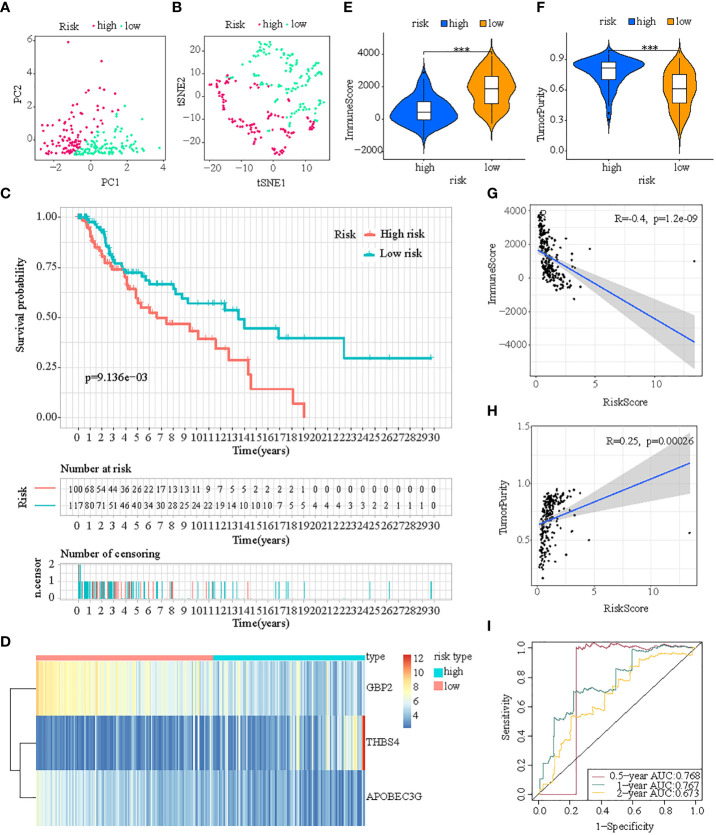
Internal verification of ICDRS in test1 cohort. **(A, B)** PCA and t-SNE analysis illustrated an excellent clustering performance of the ICDRS-based risk score; **(C)** Kaplan–Meier survival curves for the overall survival of two risk groups in the test1 cohort; **(D)** Distribution pattern of the expression levels of the 3 genes in the test1 cohort; **(E, F)** Comparing TME components in test1 cohort (*** indicates p < 0.001); **(G)** The correlation between ImmuneScore and risk score; **(H)** The correlation between TumorPurity and risk score; **(I)** AUC values of 0.5-, 1-, and 2-year in the test1 cohort.

**Figure 6:**
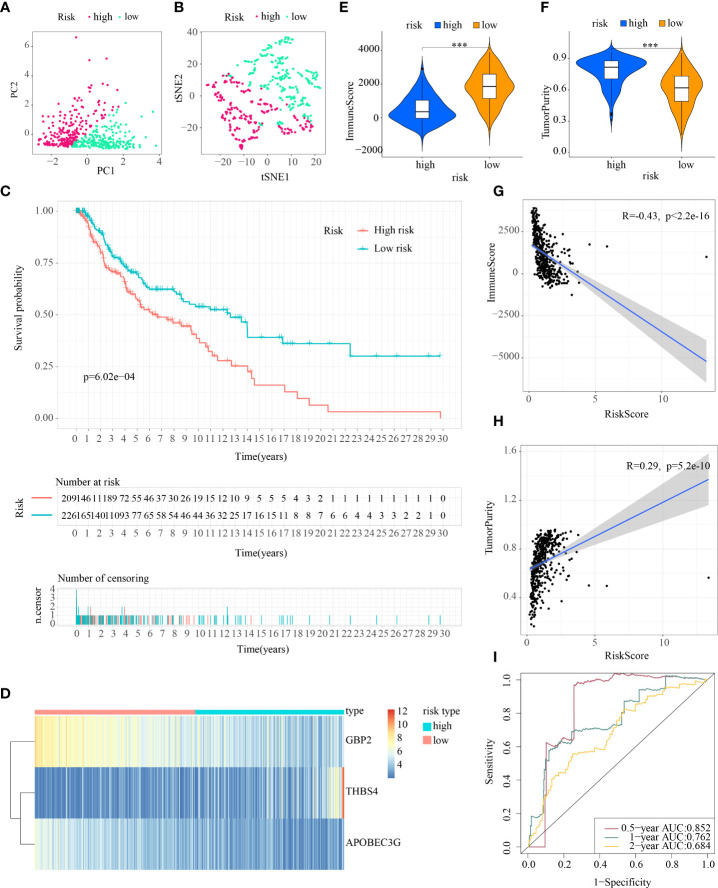
Internal verification of ICDRS in test2 cohort. **(A, B)** PCA and t-SNE analysis illustrated an excellent clustering performance of the ICDRS-based risk score; **(C)** Kaplan–Meier survival curves for the overall survival of two risk groups in the test2 cohort; **(D)** Distribution pattern of the expression levels of the 3 genes in the test2 cohort; **(E, F)** Comparing TME components in test2 cohort (*** indicates p < 0.001); **(G)** The correlation between ImmuneScore and risk score; **(H)** The correlation between TumorPurity and risk score; **(I)** AUC values of 0.5-, 1-, and 2-year in the test2 cohort.

**Figure 7:**
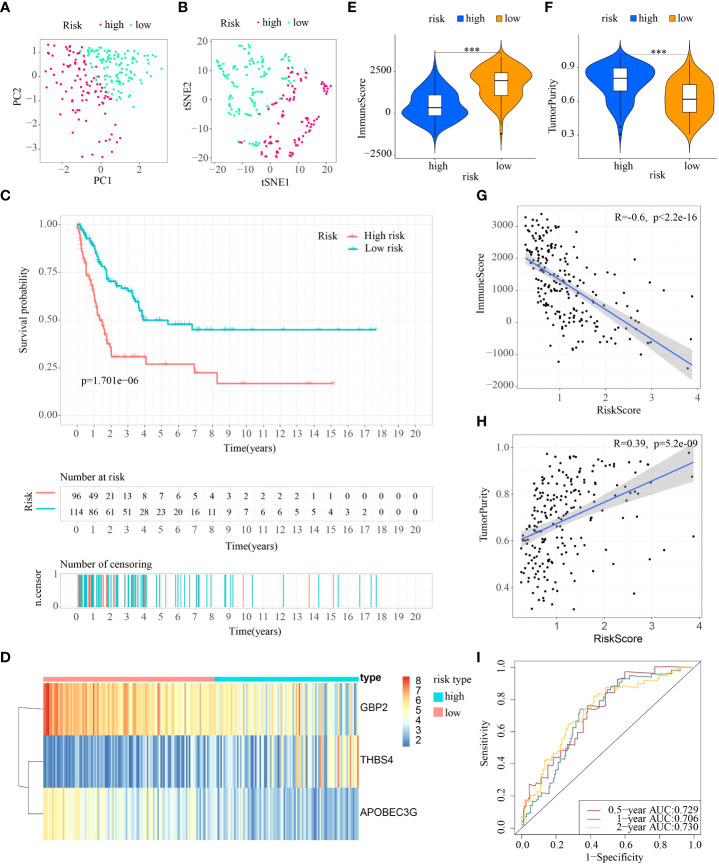
External verification of ICDRS in test3 cohort. **(A, B)** PCA and t-SNE analysis illustrated an excellent clustering performance of the ICDRS-based risk score; **(C)** Kaplan–Meier survival curves for the overall survival of two risk groups in the test3 cohort; **(D)** Distribution pattern of the expression levels of the 3 genes in the test3 cohort; **(E, F)** Comparing TME components in test3 cohort (*** indicates p < 0.001); **(G)** The correlation between ImmuneScore and risk score; **(H)** The correlation between TumorPurity and risk score; **(I)** AUC values of 0.5-, 1-, and 2-year in the test3 cohort.

First, patients in the three test cohorts were grouped respectively into low- and high-risk subpopulations according to median risk score of train cohort as the unified benchmark. For the internal validation (test 1 and test2 cohorts) and the external validation (test3 cohort), patients in the two subpopulations could be easily distinguished from one another using T-SNE and PCA (**Figures 5A, B**, **6A, B**, **7A, B**). Similarly, in the three test cohorts, patients in high-risk subpopulation experienced poorer OS (all p < 0.05) (**Figures 5C**, **6C**, and **7C**). Also, the heatmaps obtained from the three test cohorts demonstrated the presence of GBP2 and APOBEC3G with attenuated expression while THBS4 with high expression in the high-risk subpopulation (**Figures 5D**, **6D**, and **7D**). Likewise, the tumor immune microenvironment was statistically different in these three cohorts which was the same as the results in train cohort (**Figures 5E, F**, **6E, F**, and **7E, F**). Moreover, the ImmuneScore also showed a significant negative relationship with risk score in test1 cohort (R=-0.4, *p*=1.2e-09), test2 cohort (R=-0.43, *p*<2.2e-16), and test3 cohort (R=-0.6, *p*<2.2e-16) (**Figures 5G**, **6G**, and **7G**), while the TumorPurity also indicated a significant positive relationship with risk score in test1 cohort (R=0.25, *p*=0.00026), test2 cohort (R=0.29, *p*=5.2e-10), and test3 cohort (R=0.39, *p*=5.2e-09) (**Figures 5H**, **6H**, and **7H**). As for the diagnostic value of risk score, the AUC values of the ROC curves were 0.768, 0.767, and 0.673 in the test1 cohort, 0.852, 0.762, and 0.684 in the test2 cohort, and 0.729, 0.706, and 0.730 in the test3 cohort for 0.5-, 1-, and 2-year survival, respectively (**Figures 5I**, **6I**, and **7I**). Of note, all the results in the internal validation (test1 and test2 cohorts) and external validation (test3 cohorts) were consistent with those in train cohort.

**Figure 8:**
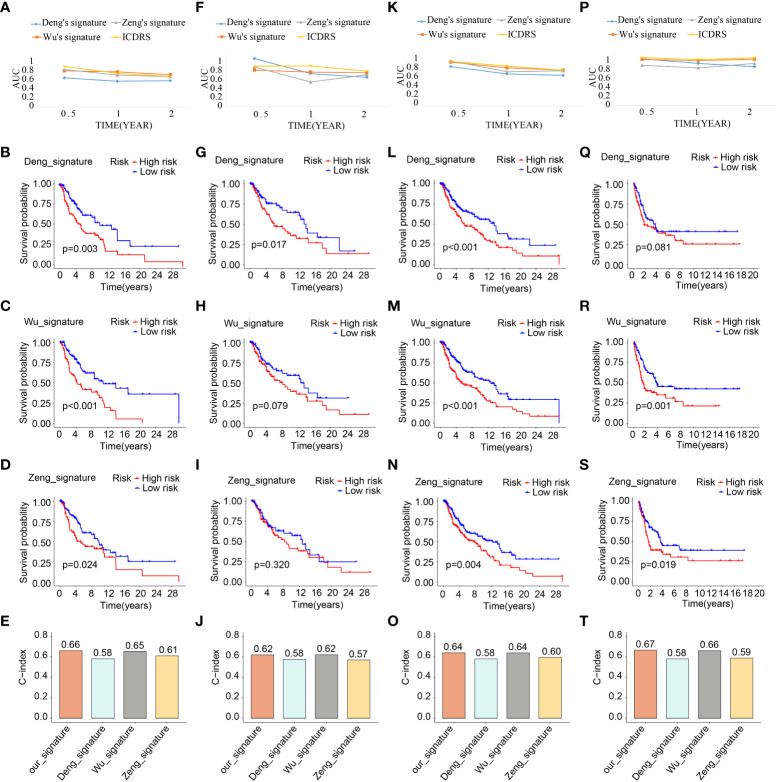
Comparative analysis of ICDRS. The comparison of AUC values, survival analysis and C-indexes between ICDRS and three additional signatures in train **(A–E)**, test1 **(F–J)**, test2 **(K–O)**, and test3 **(P–T)** cohorts.

What’s more, taking the ICDRS-based survival probability discrepancy, AUC value, and C-index into consideration simultaneously, ICDRS showed superior in prognostic value and diagnostic accuracy compared with another three signatures (**Figure 8**). On the basis of the AUC, ICDRS showed a satisfied and stable performance in all the four cohorts. Of note, the discrepancies of the survival probability in different risk subpopulations distinguished by another three signatures sometimes showed no statistical significance. And the C-indexes of ICDRS were higher than another three signatures and were 0.66, 0.62, 0.64, and 0.67 in the four different cohorts respectively.

### The ICDRS-based immune-related discrepancies in all the four cohorts

In view of the differences in tumor immune microenvironment in low- and high- risk subpopulations, more in-depth exploration of immune-related discrepancies were made in all the four cohorts in the following research.

**Figure 9:**
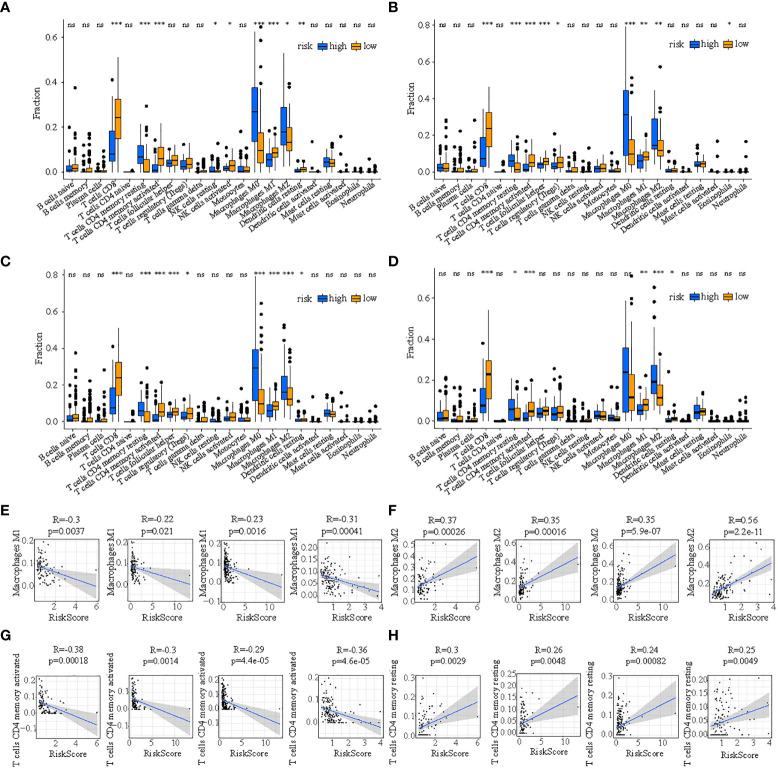
The discrepancy of tumor-infiltrating immune cells in two risk subpopulations in train **(A)**, test1 **(B)**, test2 **(C)**, and test3 **(D)** and the correlation between ICDRS-based risk score and the proportion of immune cells in the tumor immune environment in train **(E)**, test1 **(F)**, test2 **(G)**, and test3 **(H)**. (* indicates p <0.05; ** indicates p < 0.01; *** indicates p < 0.001; ns: p>0.05).

First, the consensus discrepancy of tumor-infiltrating immune cells in low- and high-risk subpopulations in the four cohorts indicated that less infiltration abundance of M1 macrophages and activated CD4+ T memory cells but more infiltration of M2 macrophages and resting CD4+ T memory cells existed in the high-risk subpopulation (**Figures 9A–D**). Subsequently, the Pearson correlation analysis showed that the proportion of M1 macrophages had a significant inverse relationship with risk score in train cohort (R=-0.3, p=0.0037), test1 cohort (R=-0.22, p=0.021), test2 cohort (R=-0.23, p=0.0016), and test3 cohort (R=-0.31, p=0.00041) (**Figure 9E**); the percentage of M2 macrophages had a significant positive relationship with risk score in train cohort (R=0.37, p=0.00026), test1 cohort (R=0.35, p=0.00016), test2 cohort (R=0.35, p=5.9e-07), and test3 cohort (R=0.56, p=2.2e-11) (**Figure 9F**); the proportion of activated CD4+ T memory cells had a significant inverse relationship with risk score in train cohort (R=-0.38, p=0.00018), test1 cohort (R=-0.3, p=0.0014), test2 cohort (R=-0.29, p=4.4e-05), and test3 cohort (R=-0.36, p=4.6e-05) (**Figure 9G**); the percentage of resting CD4+ T memory cells had a significant positive relationship with risk score in train cohort (R=0.3, p=0.0029), test1 cohort (R=0.26, p=0.0048), test2 cohort (R=0.24, p=0.00082), and test3 cohort (R=0.25, p=0.0049) (**Figure 9H**).

**Figure 10:**
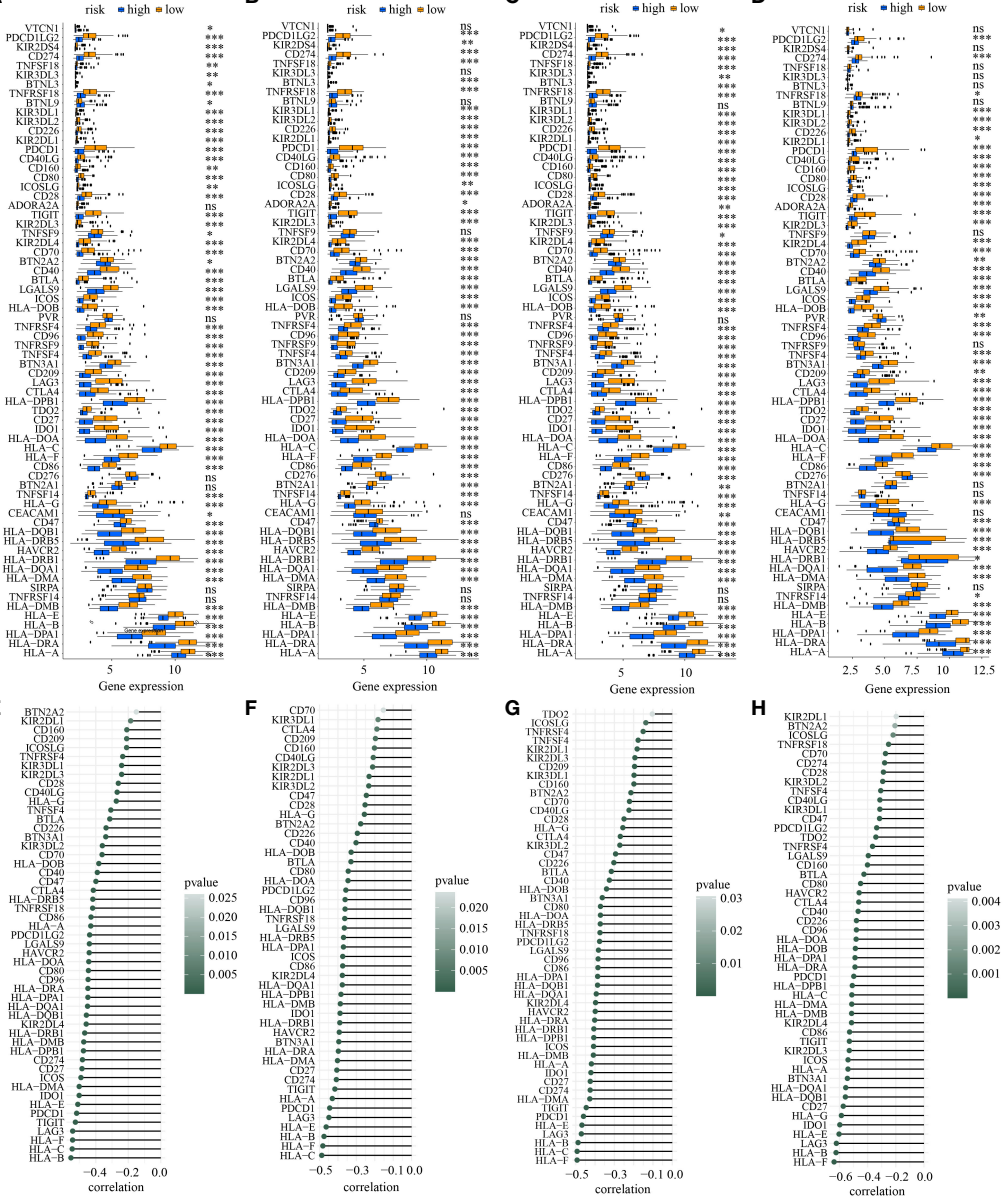
The discrepancy of the expression levels of ICGs in two risk subpopulations in train **(A)**, test1 **(B)**, test2 **(C)**, and test3 **(D)** and the correlation between ICDRS-based risk score and the expression of ICGs in train **(E)**, test1 **(F)**, test2 **(G)**, and test3 **(H)**. (* indicates p <0.05; ** indicates p < 0.01; *** indicates p < 0.001; ns: p>0.05).

Additionally, the discrepancies of ICGs’ expression in low- and high-risk subtypes in the four cohorts showed that a total of 52 ICGs had decreasing expression in high-risk subpopulations (**Figures 10A–D**). It is 30 ICGs (HLA-A, BTLA, CD80, HLA-C, CD27, CD40, CD86, BTN3A1, HLA-DMB, CD96, HAVCR2, HLA-B, HLA-DMA, ICOS, HLA-DOB, LGALS9, PDCD1, HLA-DPB1, HLA-F, HLA-DOA, HLA-DRA, HLA-E, HLA-DQA1, IDO1, KIR2DL4, LAG3, PDCD1LG2, HLA-DPA1, HLA-DQB1, and TIGIT) indicated a moderately inverse relationship with risk score in the four cohorts simultaneously (all R<-0.3, all *p*<0.05) (**Figures 10E–H**).

**Figure 11:**
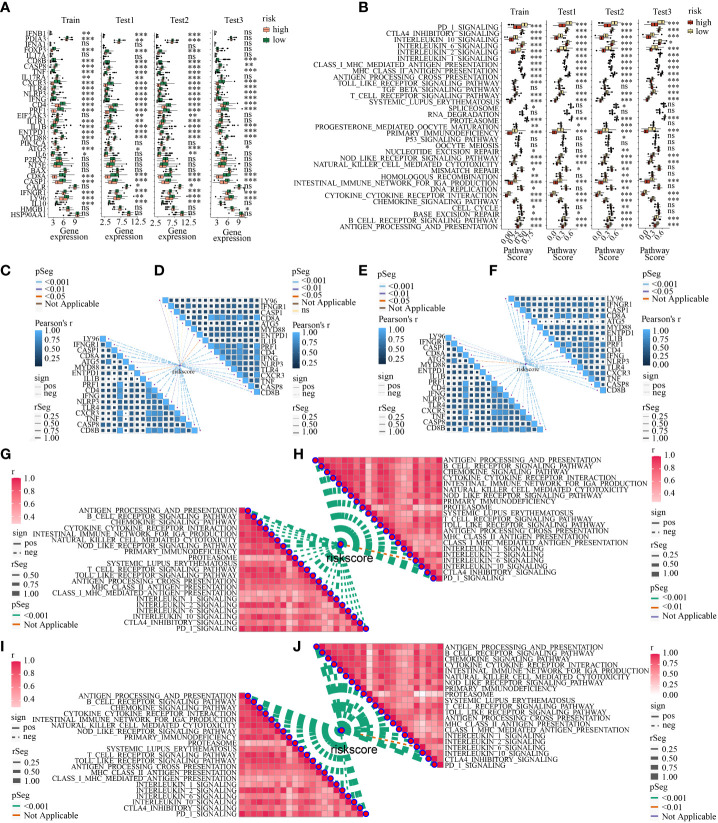
**(A)** The discrepancy of the expression levels of ICD-related genes in high- and low-risk subpopulations in train, test1, test2, and test3; **(B)** The discrepancy of the activity of the immune-related pathways in two risk subpopulations in train, test1, test2, and test3; The correlation between ICDRS-based risk score and the expression of ICD-related genes in train **(C)**, test1 **(D)**, test2 **(E)**, and test3 **(F)**; The correlation between ICDRS-based risk score and immune-related pathway scores in train **(C)**, test1 **(D)**, test2 **(E)**, and test3 **(F)**. The correlation between ICDRSbased risk score and immune-related pathway scores in train **(G)**, test1 **(H)**, test2 **(I)**, and test3 **(J)**. (* indicates p <0.05; ** indicates p < 0.01; *** indicates p < 0.001; ns: p>0.05).

Next, ICD-related genes also had differences in low- and high-risk subpopulations. A total of 17 ICD-related genes (ATG5, CASP1, CASP8, CD4, CD8A, CD8B, CXCR3, ENTPD1, IFNG, IFNGR1, IL1B, LY96, MYD88, NLRP3, PRF1, TLR4, TNF) had decreasing expression in high-risk subpopulation in all the four cohorts simultaneously (**Figure 11A**). What’s more, the activation of each immune-related pathway was different in low- and high-risk subpopulations. High-risk subtype featured a decreasing activation of immune-related pathways. There were 21 pathways showed statistical differences in the two subpopulations in the four cohorts simultaneously (**Figure 11B**). Of note, 6 ICD-related genes (CD8A, PRF1, IFNG, CXCR3, TNF, CD8B) showed a moderately negative correlation with risk score in the four cohorts consistently (all R<-0.3, all *p*<0.05) (**Figures 11C–F**). And 20 of 21 statistically different immune-related pathways (such as MHC class I and II-mediated antigen presentation and processing, Toll-like and NOD-like receptor signaling pathway, T cell and B cell receptor signaling pathway, NK cell-mediated cytotoxicity, IL-1, IL-2, and IL-10 associated signaling pathway, PD-1 and CTLA-4 associated pathways) showed a moderately negative correlation with risk score in the four cohorts similarly(all R<-0.3, all *p*<0.05) (**Figures 11G–J**).

### Prediction of immunotherapy response and potential drugs for melanoma treatment based on ICDRS

**Figure 12:**
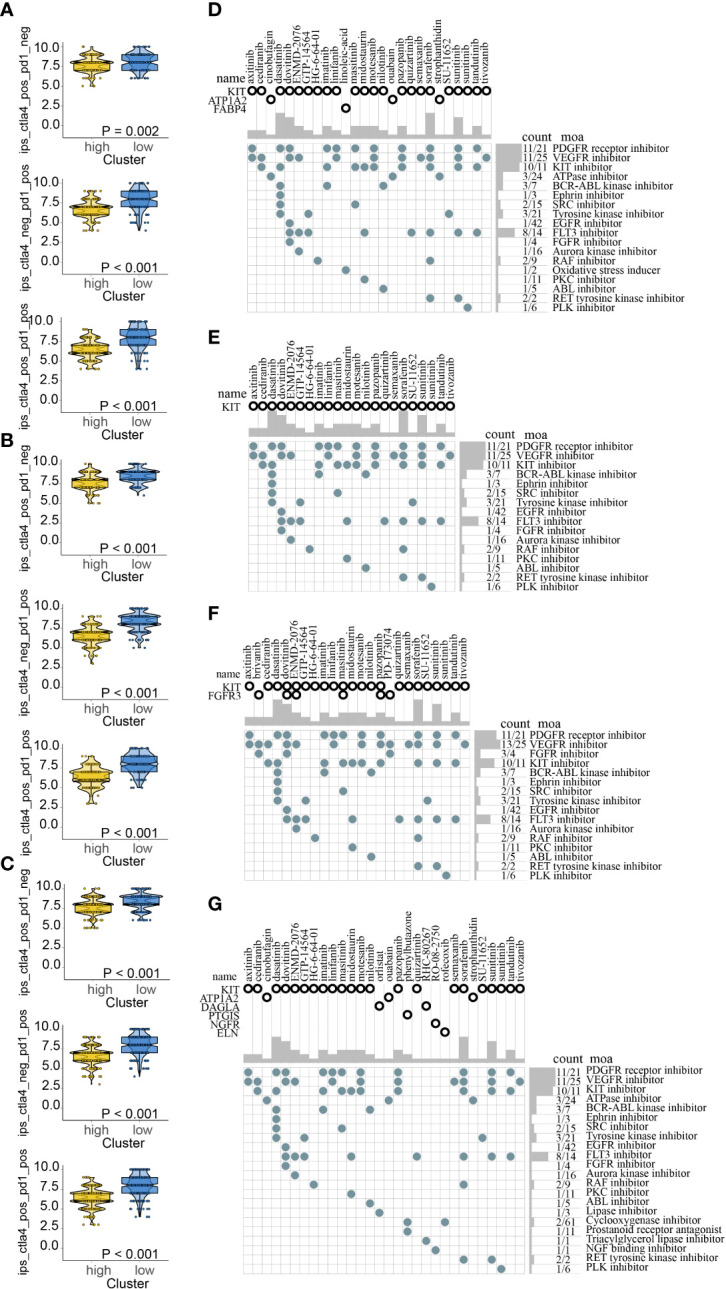
Immunotherapy response prediction in train **(A)**, test1 **(B)**, test2 **(C)**; Potential drugs targeted the upregulated DEGs in high-risk for melanoma treatment based on ICDRS in train **(D)**, test1 **(E)**, test2 **(F)**, and test3 **(G)**.

Recent researches suggested that IPS based on immunogenicity is helpful in immunotherapy response prediction. The response probabilities of using anti-PD-1 antibody and anti-CTLA-4 antibody in the different ICDRS subpopulations were analyzed. It indicated that high-risk subpolulation had lower IPS and might have a worse immunotherapy response (**Figures 12A–C**). The upregulated target DEGs in high-risk were explored with the criteria for filtering were FDR < 0.05 and log2 FC > 1 in all the four cohorts respectively. In view of respective prediction of potential drugs in the four cohorts, 31 drugs which acted on the upregulated target DEGs were as follows: axitinib, brivanib, cediranib, cinobufagin, dasatinib, dovitinib, ENMD-2076, GTP-14564, HG-6-64-01, imatinib, linifanib, masitinib, midostaurin, motesanib, nilotinib, orlistat, ouabain, pazopanib, phenylbutazone, PD-173074, quizartinib, RHC-80267, RO-08-2750, rofecoxib, semaxanib, sorafenib, strophanthidin, SU-11652, sunitinib, tandutinib, and tivozanib. And the action mechanisms were shown in **Figures 12D–G**.

## Discussion

Melanoma, the deadliest type of skin cancer, is a deadly disease that is becoming more common (48). It accounts for about 1.7 percent of all newly diagnosed primary malignant malignancies worldwide, and melanoma patients account for about 0.7 percent of all cancer deaths (49–51). Due to the influence of ICD on survival in many types of tumor including lung (52), ovarian malignancies (22), and head and neck squamous cell carcinoma (53) and cancer therapy (54–56), it is meaningful to explore whether ICD has a significant impact in tumor initiation and progression and whether ICD-related prognostic factors can be novel therapy targets in melanoma.

Hence, two subtypes (C1 and C2) were classified by NMF clustering. Based on the existence of survival and immune-related discrepancies in C1 and C2, ICD-DEGs were identified and utilized to construct a novel ICDRS. After internal and external validation, a 3-gene signature, involving GBP2, THBS4, and APOBEC3G, were unearthed. The prognostic significance of GBP2 and APOBEC3G in melanoma has also been backed up by other research investigations. It is reported that GBP2 exerted anti-tumour effects by inhibiting the Wnt/β-catenin pathway in skin cutaneous melanoma (SKCM) (57) and showed an association with poor prognosis in SKCM when its expression decreased (58). APOBEC3G, as a member of the cellular polynucleotide cytidine deaminases, catalyzes the deamination of cytosine to uracil in single-stranded DNA (59, 60) is significantly correlated with better prognosis when its expression is elevated in SKCM patients (61). As for THBS4, its potential role and prognostic performance in melanoma remains unclear but it is linked to poor prognosis in many other cancers: it effects the amplification and metastasis of gastric cancer positively (62); it may facilitate invasion of tumour cells in breast cancer (63); it accelerates HCC progression by modulating ITGB1 through FAK/PI3K/AKT pathway (64).

To guarantee the comprehensive verification and broad applicability of the prognostic signature, four cohorts (TCGA: train, test1 and test2 cohorts; GEO: test3 cohort) were identified. It’s worth noting that the internal validation cohort (test1 and test2 cohorts) and external validation cohort (test3 cohort) coexisted. It is the 3-gene signature that contributes to the differentiation of patients to low- and high-risk subpopulations. In all the four cohorts, our signature showed consistently satisfactory performance: (1) patients in the different risk subpopulations might be plainly discriminated from one another; (2) patients in the high-risk subpopulation have a dismal prognosis; (3) the signature-related tumor immune microenvironments in low- and high-risk subpopulations are statistically different; (4) ImmuneScore had a significant inverse relationship with risk score while the TumorPurity had a strong favorable relationship with risk score; (5) the diagnostic values of the signature for 0.5-year, 1-year, and 2-year survival rates were satisfactory; (6) ICDRS showed superior in prognostic value and diagnostic accuracy compared with another three well-recognized signatures.

As for the following in-depth investigation of the ICDRS-based immune-related discrepancies, it has been discovered that more infiltration of M2 macrophages but less infiltration of M1 macrophages existed in the high-risk subpopulation. Of note, proinflammatory M1 macrophages have the ability to phagocytose tumor cells, whereas anti-inflammatory M2 macrophages facilitate tumor development and invasion (65–67). Consequently, the discrepancies of these immune cell infiltration may result in a dismal prognosis of patients in high-risk subpopulation. Additionally, the inverse relationship between risk score and 30 ICGs suggest immune checkpoint inhibitor therapy could be more efficient for low-risk patients. And the negative correlation between 6 ICD-related genes (CD8A, PRF1, IFNG, CXCR3, TNF, CD8B) and risk score and the protective function of these 6 genes in SKCM indicates that drugs targeting these genes may be a novel treatment method in melanoma. Moreover, many immune-related pathways had different activities in the two subpopulations with different risk, and their activities were negatively linked with risk score. All of these differences could be the cause of differing prognoses and could be used as immunotherapy targets.

Finally, ICDRS-based immunotherapy response prediction suggested that low-risk subpopulation may benefit from anti-PD-1 and anti-CTLA-4 therapies. And the potential drugs targeted DEGs between the different risk populations were explored. Due to the increasing expression in high-risk subpopulation, these drugs might be effective for the high-risk populations.

This study has some limitations that should be acknowledged. To begin, the ICDRS was created with a small sample of melanoma patients from the TCGA and GEO databases. To confirm the predictive significance of this prognostic signature, a large-scale prospective clinical research is required. Besides, the ICDRS was generated solely through bioinformatic research, and further basic investigations are required to corroborate the conclusions.

## Conclusions

We successfully separated the TCGA-melanoma samples into two subtypes on the basis of the expression of the ICD-related genes and developed a prognostic ICDRS involving 3 genes (i.e., GBP2, THBS4, and APOBEC3G) based on the DEGs between the two subtypes. The ICDRS exhibited good diagnostic values and correlated with different tumor immune microenvironment in train cohort, internal validation cohorts (test1 and test2 cohorts) and external validation cohort (test3 cohort). As a result, the ICDRS, based on the expression of three ICD-dependent DEGs, might be applied to determine the prognosis, the infiltration of M1/M2 macrophages, the expression levels of ICGs and ICD-related genes, as well as the functioning of immune-related pathways in melanoma. This will aid in patient classification for tailored melanoma treatment.

## Data availability statement

The datasets presented in this study can be found in online repositories. The names of the repository/repositories and accession number(s) can be found in the article/**Supplementary Material**.

## Author contributions

The content and authorship of the paper are solely the responsibility of the authors. JR, JY, and SN contributed to the design of the research, the collecting and analysis of data, and the preparation and review of the paper. YW is tasked with data collecting. JW, LZ, and JL reviewed/edited the manuscript. All authors contributed to the article and approved the submitted version.

## Funding

The research was conducted with support from the National Natural Science Foundation of China (No. 82172793).

## Conflict of interest

The authors declare that the research was conducted in the absence of any commercial or financial relationships that could be construed as a potential conflict of interest.

## Publisher’s note

All claims expressed in this article are solely those of the authors and do not necessarily represent those of their affiliated organizations, or those of the publisher, the editors and the reviewers. Any product that may be evaluated in this article, or claim that may be made by its manufacturer, is not guaranteed or endorsed by the publisher.
