# Supplementary figures and images for: Comprehensive characterisation of immunogenic cell death in melanoma revealing the association with prognosis and tumor immune microenvironment
Source: Front Immunol. 2022 Sep 23;13:998653. doi: 10.3389/fimmu.2022.998653 (PMC9538190; doi:10.3389/fimmu.2022.998653)

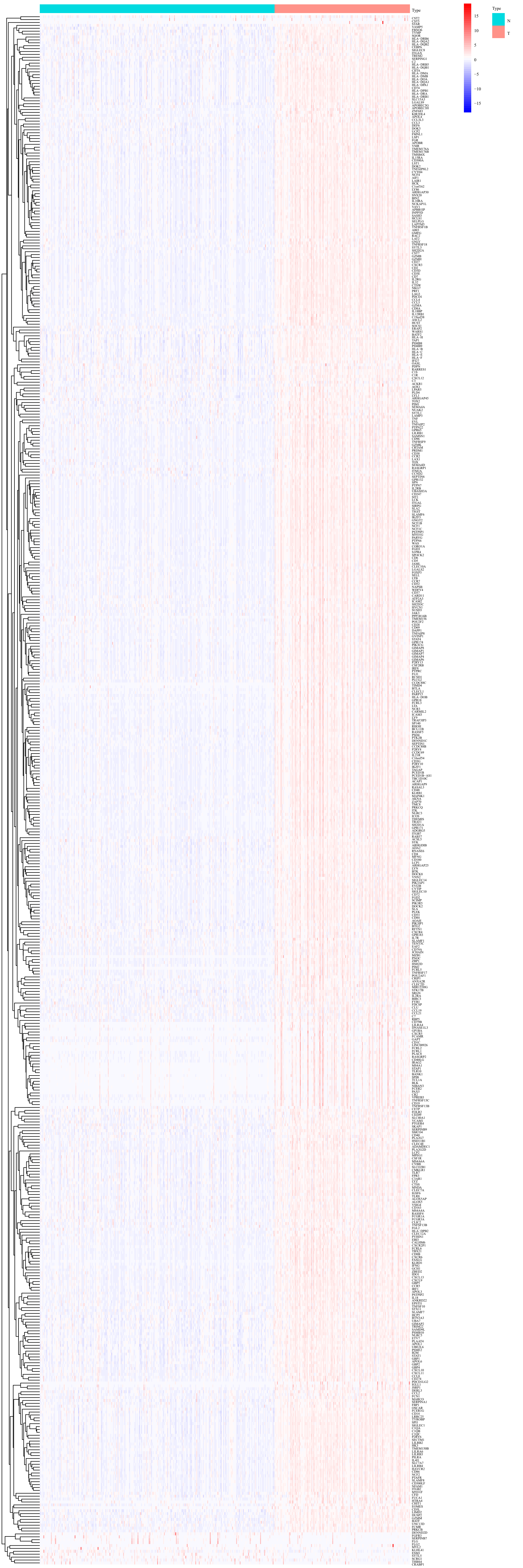

Supplement: Supplementary file 1 [file DataSheet_1.pdf]

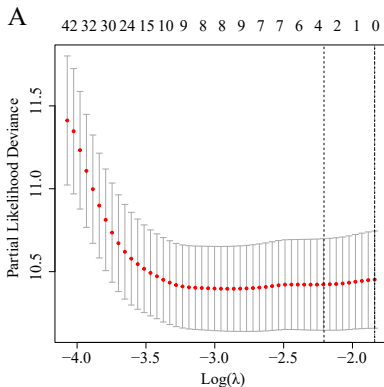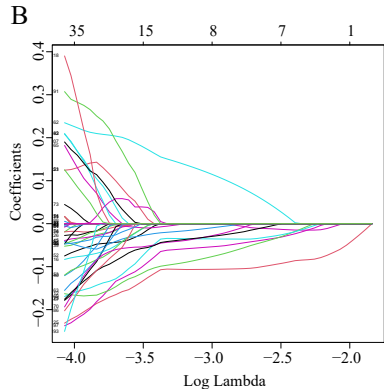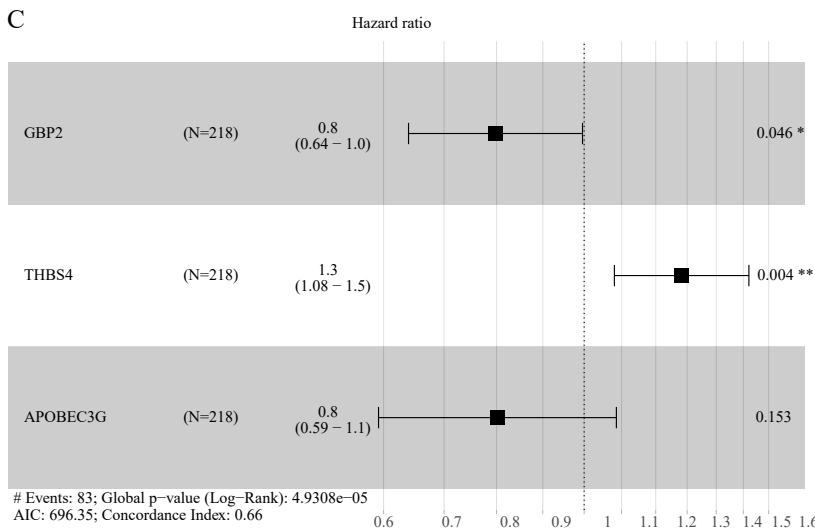

Supplement: Supplementary file 2 [file DataSheet_2.pdf]
